# Design Considerations for a Sub-mW Wireless Medical Body-Area Network Receiver Front End

**DOI:** 10.3390/mi9010031

**Published:** 2018-01-17

**Authors:** Ehsan Kargaran, Danilo Manstretta, Rinaldo Castello

**Affiliations:** Department of Electrical, Computer and Biomedical Engineering, University of Pavia, 27100 Pavia, Italy; danilo.manstretta@unipv.it (D.M.); rinaldo.castello@unipv.it (R.C.)

**Keywords:** wireless body area network, ultra-low-power, ultra-low-voltage, receiver, low-noise-amplifier

## Abstract

Wireless medical body-area networks are used to connect sensor nodes that monitor vital parameters. The radio consumes a large portion of the sensor energy budget, and hence its power dissipation should be minimized. The low-noise amplifier (LNA) is an important component of the receiver, and must guarantee low-noise amplification and impedance matching. In this work, an ultra-low-voltage ultra-low-power LNA is proposed that, thanks to the proposed transformer-based gate boosting technique, has a reduced current consumption of only 160 μA and can operate with a supply as low as 0.18 V. The LNA was designed using 40 nm Complementary Metal-Oxide Semiconductor (CMOS) technology and features a voltage gain of 14 dB, 5.2 dB NF and −8.6 dBm IIP3. This performance is comparable to a prior work by the same authors, but with the minimum supply voltage reduced by a factor of 4x.

## 1. Introduction

Wearable wireless medical devices for continuous health monitoring are an important asset in present and future medical assistance. They can be of great benefit as a prevention tool for early detection of anomalous conditions in the general population, as well as a part of rehabilitation after surgical or other major medical procedures or as part of the standard diagnostic procedure and treatment of chronic diseases. Low-power Wireless Body-Area Networks (WBANs) offer an optimal solution for the interconnection of medical devices on, in, or around the human body. The IEEE 802.15.6 standard provides the system specifications for low-power, short-range, highly reliable radio links [1]. Reliability and small form factor are key enabling factors for the widespread adoption of these devices. To ensure both, it is important to have radios that dissipate low power while meeting standard specifications with sufficient margin, in order to provide tolerance for unfavorable wireless link conditions. In wearable devices, the radio typically consumes most of the energy [2,3]. In a typical WBAN radio, both transmitter and receiver consume a few mWs [2,4,5,6,7,8,9,10,11]. However, while the transmitter is typically operated with a very low duty cycle, since data transmission is operated in bursts, the receiver is continuously operating, and is therefore the element primarily responsible for the average power dissipation [12]. In the receiver, the most power-hungry components are the low-noise amplifier (LNA) and the frequency synthesizer, in particular the oscillator and frequency dividers. When using energy coming from a small single-cell battery, wirelessly transmitted [13] or scavenged from the environment [14], the use of a low supply voltage is preferred [15]. Scaling down the supply voltage well below 1 V is very beneficial to lowering power dissipation, especially in the Voltage Control Oscillator (VCO) and frequency dividers, where power scales down with the square of the supply voltage. In the LNA, on the other hand, the current consumption does not scale down with the supply since it is limited by its design constraints. In this work, we address the problem of reducing the power dissipation of the LNA from around one mW down to 30 μW, while still meeting the basic LNA requirements of impedance matching, acceptable noise, gain and linearity. The paper is structured as follows: Section 2 provides the system overview and derives the receiver requirements, Section 3 reports the circuit design and analysis, Section 4 summarizes the simulation results, and Section 5 draws the conclusions.

## 2. System Overview 

The main communication standards for short-range and low-power applications are IEEE 802.15.4, IEEE 802.15.6, and Bluetooth Low Energy (BLE). Among these, IEEE 802.15.6 is dedicated to wireless medical body-area networks (MBAN). The US MBAN operates in the 2.4 GHz ISM band ranges between 2.36 and 2.4 GHz, and the Federal Communication Commission (FCC) Part 15 unlicensed 2.4 GHz ranges from 2.4 to 2.5835 GHz, providing 118 channels across both bands. The standard specifies differential quadrature phase-shift keying (DQPSK) operating at a symbol rate of 600 ks/s. The sensitivity of −92 dBm is based on a data rate of 971.4 kb/s, and the required Signal to Noise Ratio (SNR) is 11.2 dB, leading to a maximum Noise Figure (NF) of 19.2 dB for the entire receiver. Moreover, the relaxed adjacent channel rejection ratio (ACRR) of only 9 dB leads, according to the derivation in [16], to the required adjacent channel IIP3 of −55 dBm, which is a very relaxed specification. On the other hand, in order to withstand larger out-of-band blockers, an out-of-band (OOB) IIP3 of −19 dBm is required [16]. 

Therefore, the requirements of the IEEE 802.15.6 standard (in terms of noise and linearity) are very easy to meet. To take advantage of such relaxed requirements in MBAN applications, designing a receiver with ultra-low power consumption is crucial. The first active component of the receiver is the Low Noise Amplifier (LNA), and this can generally be considered to be the most challenging and power-hungry component, due to its performing several significant tasks simultaneously. Firstly, input power matching the 50 Ω source impedance has to be preserved not only to maximize the available power, but also to guarantee proper operation of the external Surface Acoustic Wave (SAW) filters. Secondly, it must add as little noise as possible. Thirdly, the input signal must be amplified to reduce the noise contribution of the subsequent stages in the receiver. Finally, in the receiver chain, the LNA most likely limits the OOB IIP3, and its value has to be at an acceptable level.

## 3. Circuit Description

### 3.1. Passive Gain Boosting

**Figure 1:**
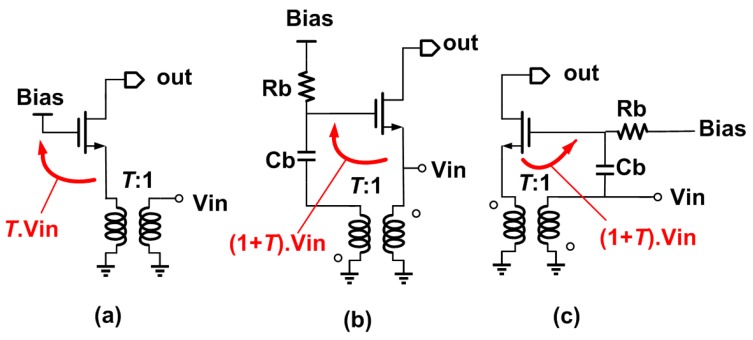
Passive *g_m_* boosting common-gate (CG) amplifiers (**a**–**c**).

A simplified schematic, shown in Figure 1a, can be used to start describing the proposed common-gate (CG) LNA. In a basic CG LNA, the current consumption is constrained by the input device *g_m_*, which is set to 20 mS to perform input power matching. Two techniques can be used to lower the device *g_m_* and hence the power dissipation: source impedance boosting and gate boosting. If a 1:*T* transformer is introduced in front of the CG amplifier, as shown in Figure 1a, the source impedance is boosted by a factor *T*^2^. This lowers the device *g_m_*—and hence the current—by the same factor. The same could be achieved using an LC matching network, but with narrower bandwidth and increased sensitivity to inductor losses. If the 1:*T* transformer is introduced between the source and gate of the input transistor, as shown in Figure 1b, the gate-source voltage is boosted by a factor 1 + *T* with respect to the input voltage, and the device *g_m_* can be reduced by the same factor. 

Merging the impedance transformation and passive gain boosting is the basic idea of the proposed LNA, as shown in Figure 1c.

The primary of the transformer is connected to the input of the LNA and is AC-coupled to the gate of the input device, while the secondary is directly connected to the source of the input device. Assuming (ideally) a transformer with *k* = 1, the source voltage is scaled up by a factor of *T*, while the gate-source voltage is enhanced by a factor 1 + *T* with respect to the input. The *G_m_* is therefore:

(1) $$G_{m}=\left(1+T\right)g_{m}$$

The input impedance of the proposed passive gain boosting can be computed as follows:

(2) $$Z_{in}=\frac{1}{g_{m}T(1+T)}$$

**Figure 2:**
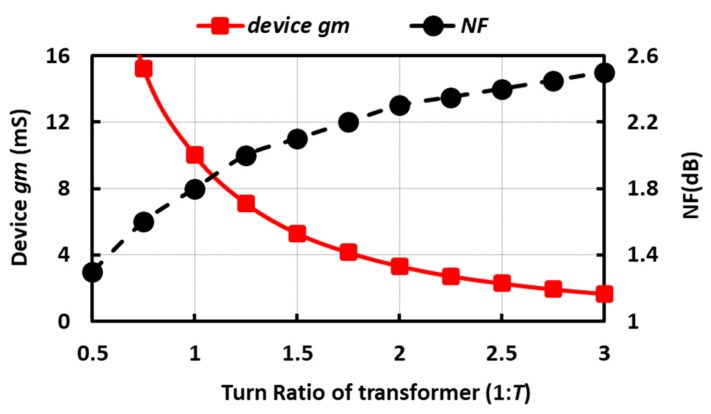
Simulation of required device *g_m_* and NF vs. *T*.

Taking the power-matching condition as a constraint, the schematic in Figure 1c with lossless transformer is simulated to provide an estimation of the required device *g_m_* and the feasible NF as a function of transformer turns ratio; the results are shown in Figure 2. Although a higher signal current and improved NF can be achieved using a step-down transformer (*T* < 1), it considerably increases the power consumption. For instance, NF of 0.8 dB can be achieved by choosing *T* = 0.25; however, this requires the device *g_m_* of 64 mS to perform input power matching, which is tremendously power hungry for this application. Where *T* = 1, the device *g_m_* has to be 1/(2*R_S_*) for input power matching, and *G_m_* is the same as with a simple CG amplifier that carries twice as much current (i.e., doubling the *G_m_/I_d_* ratio). Where *T* = 2, the device *g_m_* only has to be 1/(6*R_S_*) to perform input matching, and *G_m_* is one half that of a CG amplifier carrying six times the current (i.e., it is three times as efficient). While transformers with k factors close to 0.9 can be designed, the losses of the transformer can strongly affect the NF of a LNA.

Modelling transformer losses as a resistance *R_loss_* at its secondary, the LNA noise factor is:

(3) $$F=1+\frac{\gamma T}{1+T}+\frac{T^{2}R_{S}}{R_{loss}}$$

where *γ* is the MOSFET noise parameter. Assuming a lossless transformer, for *T* = 1, *F* = 1 + *γ*/2; for *T* = 2, *F* = 1 + (2/3)*γ* and if *T* >> 1, F eventually converges to 1 + *γ* (i.e., usually less than 3 dB), while the required device *g_m_* (and hence power consumption) scales down as *T^2^*. Two issues should be taken into consideration for large *T*. Firstly, the noise contribution of the transformer is not negligible, since *R_loss_* does not scale up as *T^2^*. Secondly, the *G_m_* scales down as 1/*T*, making the noise of the subsequent stages in the receiver chain to be more important. Hence, *T* = 2 is chosen, and to minimize further power, additional power-saving techniques are highly desirable. 

**Figure 3:**
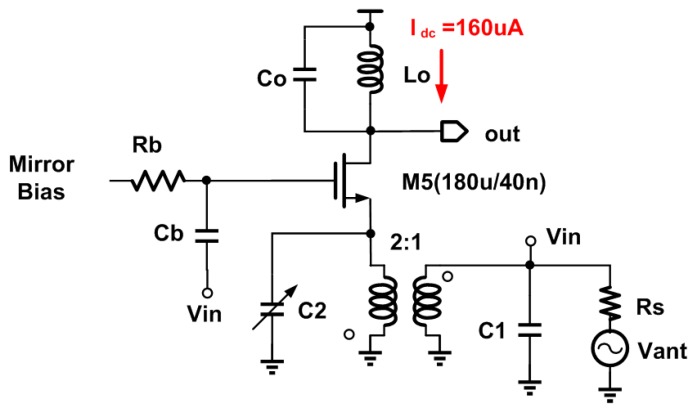
Schematic of ultra-low voltage (ULV) low-noise amplifier (LNA).

In [17], a highly efficient ultra-low power (ULP) LNA using the current reuse approach was proposed. Even though the power consumption is remarkably low (only 30 μW), due to the staking of several devices, the required supply voltage is at least 0.8 V. This is an acceptable limitation for some applications, but for MBAN, a lower supply is desirable. Thus, using ultra-low supply voltage as an alternative power-saving technique is proposed here. To consume the same power as in [17], the supply voltage is lowered to 0.18 V. This leaves a sufficient drain-to-source voltage drop in the input device to operate in the saturation region [17]. Furthermore, based on Equation (2), the device *g_m_* needs to be 1/(6*R_S_*) to perform impedance matching considering transformer turns ratio *T* = 2. The dissipated power of ultra-low voltage (ULV) LNA, shown in Figure 3, is almost equal to that in [17], while its bias current is four times higher. Employing inductive load is inevitable, owing to the drastically restricted available voltage headroom. Additionally, to drive the gate of the transistor, the ultra-low available supply voltage has to be boosted to the required value using an ULP charge pump (e.g., in [18], the supply voltage of 0.18 V was boosted by factor of 3, reaching 0.54 V). Since a negligible static current is needed from the boosted voltage, the dissipated power and occupied area of the charge pump will be determined by other receiver building blocks, and is not further investigated here. A parallel resistor, *R_load_*, can be considered to model the noise of load inductor, and the equivalent noise factor can be given as follows:

(4) $$F=1+\frac{\gamma T}{1+T}+\frac{T^{2}R_{S}}{R_{loss}}+\frac{4T^{2}R_{S}}{R_{load}}$$

where *R_load_* is the equivalent loss of the load inductor. As can be clearly seen from Equation (4), due to the limited Quality factor (*Q*) of on-chip inductors, inductive load also contributes to the total noise factor. In fact, assuming equal loss resistance for transformer and load inductor, the noise of the load goes directly to the output, while only half of the input transformer noise current goes to the output due to the input matching, and as a result, the noise of the load counts 4 times more. The load inductor is chosen to be 3.5 nH, and has a *Q* of 11.5. It is implemented in 4 turns, with a winding width of 6 μm, a spacing of 2 μm, and an occupied area of 0.048 mm^2^. 

### 3.2. Stability Analysis

**Figure 4:**
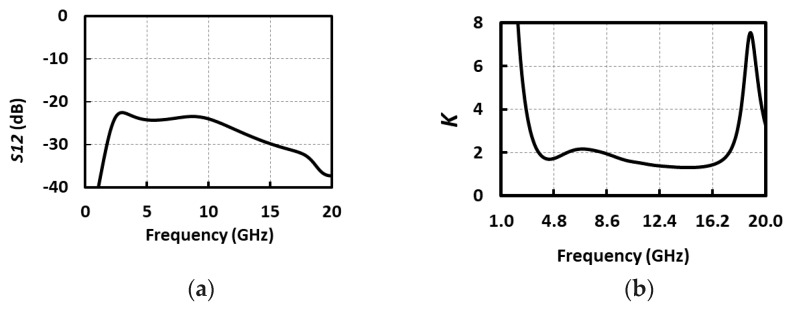
Stability simulations: (**a**) *S*_12_ and (**b**) *K* factor.

The stability of the high-frequency amplifier is a key factor, and needs to be carefully taken into account. Simple CG amplifiers are well known for their internet stability; however, due to *g_m_* boosting being employed in our design, it is necessary to investigate its stability. The load impedance can be reflected to the input due to the high-frequency feedback path introduced by the parasitic gate-drain capacitance, and this can cause stability concerns. This effect is even more disturbing for inductive loads. In fact, negative impedance can be created at the input by the inductive load because of the Miller multiplication, thus causing potential instability. Stability can be studied using Rollett’s stability factor (*K* factor), defined as [19]:

(5) $$K=\frac{1-{\left|S_{11}\right|}^{2}-{\left|S_{22}\right|}^{2}+{\left|S_{11}S_{22}-S_{12}S_{21}\right|}^{2}}{2\left|S_{12}S_{21}\right|}$$

when *K* > 1 the circuit is unconditionally stable. As can be seen in Figure 4, the ULV LNA is unconditionally stable at all frequencies. 

### 3.3. Transformer Design and Optimization

The transformer plays an important role at the LNA input, since it directly adds its noise (loss) to the antenna noise source and degrades the NF. Hence its *Q* has to be maximized. Additionally, it provides Electrostatic discharge (ESD) protection at the input, as well as boosting input transistor source voltage by a factor of 2 in this design. Due to being the only ultra-thick metal available in the 40 nm CMOS technology, it is necessary to choose a proper configuration. Although a high coupling factor can be achieved with a stacked configuration, the latter suffers from strong capacitive coupling between primary and secondary, and also gives an unequal *Q* between primary and secondary. On the other hand, much lower capacitive coupling is expected with a coplanar configuration, leading to higher and more balanced *Q* on both primary and secondary, even though it has a lower coupling factor. Both primary and secondary losses should be minimized, since large parasitic capacitances are present at both terminals. Thus, a coplanar configuration is chosen. 

**Figure 5:**
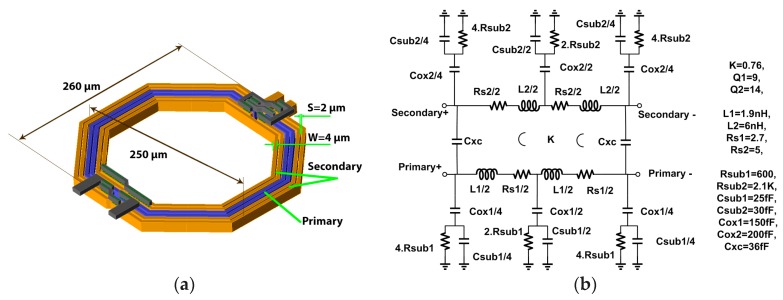
(**a**) Layout of transformer and (**b**) Lumped model.

**Figure 6:**
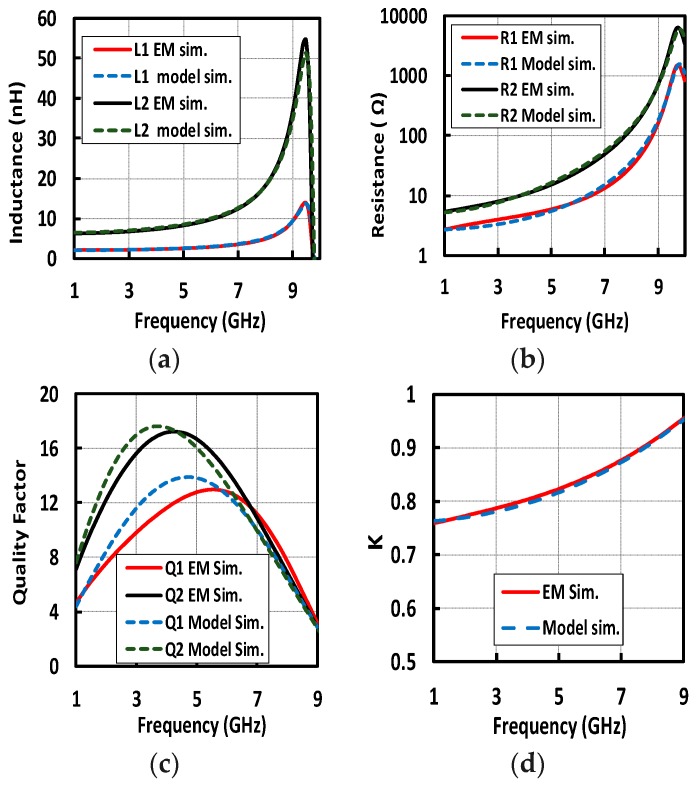
Simulation results of EM and lumped model of transformer, (**a**) inductance, (**b**) loss, (**c**) Quality factor, (**d**) coupling factor.

To design an efficient transformer for the LNA in Figure 3, the following aspects have to be taken into the account. Transformer losses can be modelled simply as a parallel loss resistance, proportional to *L* and *Q*, that needs to be maximized. A large number of turns, narrow spacing and large radius can maximize the inductance value. Increasing the radius, winding width, and winding spacing results in improved *Q*. Finally, reducing inter-winding spacing and increasing the number of turns can enhance the *K*. Our target is to design a step-up transformer (1:2) with the highest *Q* and *K*. The primary and secondary windings have two and four turns, respectively. Although *Q* can be improved by increasing winding width (up to the point where parallel losses linked with the substrate become dominant [19,20]), in a coplanar configuration, this degrades *K*. In a step-up transformer, parallel loss is crucial, particularly in the secondary; as a result, a winding width of 4 μm is chosen. Additionally, high inductance can be obtained by choosing a large radius; 90 μm in this design. For a given winding inductance, choosing a larger radius reduces the required number of windings, which can help reduce the parasitic capacitance and improve the *Q*. Moreover, a minimum winding width of 2 μm is selected to maximize *K*. The designed transformer has an area of 0.065 mm^2^, and is shown in Figure 5. To maximize the coupling factor, the primary made of the two middle windings is inserted between the inner and outer secondary windings. In order to accurately model and optimize transformer characteristics, EMX software (Integrand Software, Inc., Berkeley Heights, NJ, USA) was utilized to perform an Electromagnetic (EM) simulation. According to the EM simulation, a lumped model was derived to characterize the transformer, including self-inductance (*L*), quality factor (*Q*), coupling coefficient (*K*). As can be seen in Figure 6, there is a very good agreement between EM simulations and the extracted lumped model, which allows the transformer design and the trade-offs between reduced losses, maximized coupling factor and minimized area to be examined. A *Q* of 9 and 14 is achieved for the primary and secondary, respectively, and the coupling factor is close to 0.8 at 2.4 GHz. The step-up coplanar configuration exhibits higher *Q* on the secondary due to the higher self-inductance. The self-resonance occurs above 9 GHz, and the peaks of the *Q* are between 4 and 6 GHz, which shows that substrate losses were properly minimized, and ensures that high *Q* is achieved even in the worst-case corner with a low self-resonance frequency.

## 4. Simulation Results

**Figure 7:**
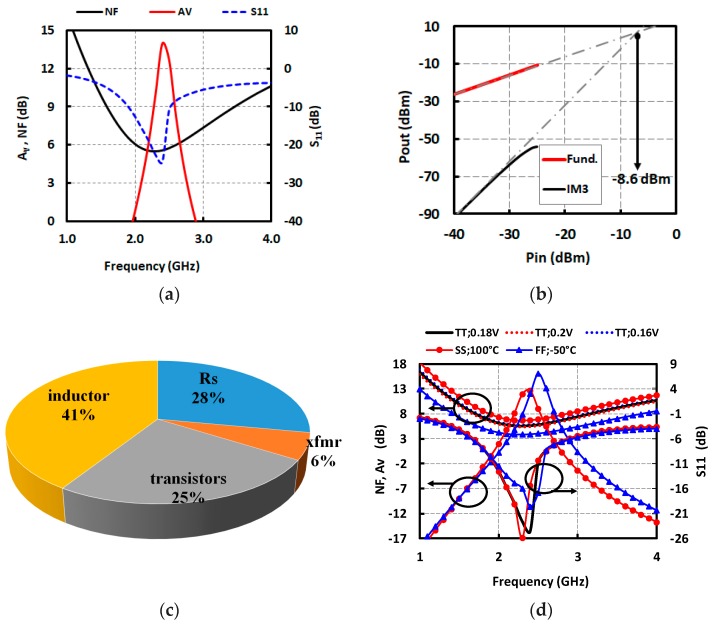

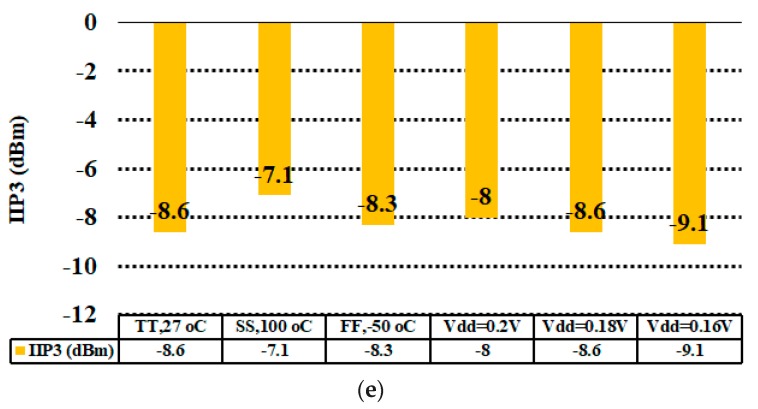
Simulation results of Electromagnetic (EM) and the lumped model of the transformer, (**a**) Voltage gain, NF and *S*_11_, (**b**) IIP3, (**c**) Noise contributors in %, (**d**) Corner simulations of ULV LNA, (**e**) IIP3 with respect to PVT variation.

The proposed LNA was designed and simulated in TSMC 40 nm CMOS technology using low-threshold devices. The ULV LNA has a supply voltage of 0.18 V, which can be generated directly from energy harvested from the environment [18]. The dissipated power of the proposed LNA is only 30 μW, excluding the biasing network. The LNA was designed and optimized to operate at 2.4 GHz. The operating frequency band can be easily tuned through a variable capacitor (C_2_) placed on the secondary of the transformer, and also by means of the load capacitor. The performance of the proposed ULV LNA when it is tuned to operate at 2.4 GHz for WBAN applications is shown in Figure 7. A well-matched input impedance (*S*_11_ = −25 dB) is preserved at the desired frequency and voltage gain, and an NF of 14 dB and 5.2 dB, respectively, is obtained at the desired frequency. To simulate IIP3, two input tones are placed around 2.4 GHz with 20 MHz offset. As shown in Figure 7b, the simulated IIP3 is around −8.6 dBm.

Figure 7c shows the noise contribution of the LNA. Simulations do not include the noise contribution from the voltage regulator that would be required in real applications. As can be clearly seen, the noise contribution of the transformer losses is less than 10% of the total noise, demonstrating the effectiveness of the transformer design optimization. As can be seen in Figure 7c, the predominant noise source contributor is the loss of the load inductor due to the limited *Q* of the on-chip inductor. This is due to the fact that the load inductor losses weigh 4 times more than those of the input transformer, as indicated by Equation (4). On the other hand, the load inductor enables the use of a drastically reduced supply voltage, and hence a much lower power dissipation.

The sensitivity of the proposed LNA performance with respect to the variations in process corners and supply voltage was carefully evaluated. Figure 7d depicts the effect of a supply voltage variation of ±10% on voltage gain (*A_V_*) and NF. The ULV LNA *A_V_* is almost insensitive to variations in the low (0.18 V) supply, since its gate bias voltage is generated from a separate (boosted) supply. The NF variation is less than 0.2 dB. Gain, noise and input return loss simulations are reported in three extreme process corner cases (SS @ +100 °C, TT @ 27 °C, FF @ −55 °C), and the results are shown in Figure 7. Variation of NF and gain across the process corners are ±1 dB and ±2 dB, respectively. Very good impedance matching is also preserved across process corners. Figure 7e depicts the variation of IIP3 across Process, Voltage, Temperature (PVT). Less than 1 dB IIP3 degradation with respect to the nominal value is observed in the worst-case corner. The overall sensitivity of the LNA to the supply and process corner and temperature variations is more than acceptable. Even though an experimental validation is not available at the time of this writing, the simulation results reported above emphasize the robustness of the proposed solution. These simulations were carried out using 40 nm CMOS design kit models, with over a decade of maturity, providing a high degree of confidence in the simulation results. Additionally, the losses of the transformer have been carefully evaluated though EMX electromagnetic simulations, and are fully taken into account in the circuit simulations. 

The overall performance of the proposed LNAs is compared with that of the recently published ULP LNAs in Table 1. The proposed LNA consumes much less power compared to state-of-the-art LNAs [21,22,23], and far exceeds the requirements of the intended application [16]. The ULV LNA has a NF comparable to other ULV designs (with supply below 0.5 V). Using very advanced technology of 16 nm FinFET, the LNA in [24] is supplied with only 100 mV and shows an NF of 3 dB, and it consumes just 44 μW. Nonetheless, the dissipated power of the proposed LNA is 33% lower, and its voltage gain and IIP3 are higher. Compared with [25], which is the second-lowest power LNA reported, our designs exhibit equal NF, better IIP3 and half the dissipated power. To evaluate the overall performance of the proposed LNAs, we use a classic figure of merit (FOM), defined as:

(6) $$FOM=\frac{IIP3\left(mW\right)Gain\left(lin\right)}{\left(F-1\right)Pdc\left(mW\right)}$$

The proposed design has the highest Figure of Merit (FOM) among all previously published works reported in Table 1, except for our prior work [23], which requires a supply voltage that is 4 times higher. Since an ultra-low supply voltage is required for WBAN applications, the proposed ULV LNA demonstrates an overall competitive performance. For fair comparison, it should be mentioned that while [24,25,26,27] report measured results, this work, together with [17,21,22,23] only report simulation results.

## 5. Conclusions

The design methodology and optimization of an ultra-low voltage LNA was presented in this paper. Thanks to the significantly reduced supply voltage and the utilization of transformer-based passive gain boosting, the power consumption of the proposed LNA is as low as 30 μW, while it operates with only 0.18 V supply voltage. The simulation results in 40 nm CMOS technology validate the LNA’s functionality with respect to PVT variation, and it shows an NF of 5.2 dB and a voltage gain of 14 dB at the desired frequency. The proposed LNA is suitable for wireless medical body-area networks where the power budget is heavily reduced.

## Figures and Tables

**Table 1:** Performance summary and comparison with state-of-the-art LNAs.

**References**	ULV LNA	[17]	[21]	[22]	[23]	[24]	[25]	[26]	[27]
**Freq (GHz)**	2.4	2.4	2.14	5	0.1–1.6	2.4	2.4	0.6–3.1	1
**Tech (nm)**	40	40	65	180	90	16	130	130	130
**Vdd (V)**	0.18	0.8	0.6	0.6	1	0.1	0.4	0.4	1
**Pdc (μW)**	30	30	402	1300	425	44	60	160	100
**NF (dB)**	5.2	3.3	2.8	3.5	5.5	3	5.3	4.5	3.9
**Gain (dB)**	14	14.2	9.2	12.5	10.5	10.8	13.1	13	16.9
**IIP3 (dBm)**	−8.6	−11.6	NA	−2	−4.5	−18	−12.2	−12	−11.2
**FOM**	10	10.4	NA	2.11	1.1	1.58	1.9	0.97	3.65
**S/M**	S	S	S	S	S	M	M	M	M

S/M: Simulation/Measurement.
